# TNF-induced chronic inflammation does not affect tumorigenesis driven by p53 loss

**DOI:** 10.1038/cddis.2016.470

**Published:** 2017-01-12

**Authors:** Derek Lacey, Andreas Strasser, Philippe Bouillet

**Affiliations:** 1 The Walter and Eliza Hall Institute of Medical Research, Parkville, Melbourne, VIC, Australia; 2 Department of Medical Biology, The University of Melbourne, Melbourne, Melbourne, VIC, Australia

*Dear Editor*,

A growing body of epidemiological and clinical data support the concept that chronic inflammation promotes tumour initiation and progression. There is also substantial evidence of a mutual negative regulation between p53, a major tumour suppressor, and NF-*κ*B, a major regulator of inflammation.^1^

The tumour suppressor gene *TP53* is the most frequently mutated gene (~50% of all tumours) in human cancer.^2^ Accordingly, mice deficient for p53 (*TP53*^*−/−*^) develop malignancies early in life, and on a *C*57BL/6 background most die between 100–250 days from thymic lymphomas. Heterozygous *TP53*^+/−^ mutant mice also develop tumours, but these occur much later in life (400–700 days), and most are sarcomas, haemangiomas and lymphomas.^3^

BPSM1 (bone phenotype spontaneous mutant 1) mice develop severe arthritis and heart disease as a result of a transposon insertion into the 3′untranslated region (3′UTR) of the *Tnf* gene; this dominant mutation causes marked overexpression of this major inflammatory cytokine.^4^ Recent reports have suggested that TNF is involved in all aspects of carcinogenesis: cellular transformation, survival, proliferation, invasion, angiogenesis, and metastasis.^5, 6^ To test whether the chronic inflammation associated with constitutive TNF overexpression would accelerate tumorigenesis, we crossed *p53*-deficient mice with BPSM1 mice, and recorded the development of tumours and/or paralysis (due to rheumatoid arthritis) in *BPSM1*^*+/+*^ *TP53*^*−/−*^*, BPSM1*^*m/+*^ *TP53*^*−/−*^, *BPSM1*^*m/+*^ *TP53*^*+/+*^, and *BPSM1*^*m/+*^ *TP53*^*+*/−^ mice. *BPSM1*^*m/m*^ mice rarely live longer than 7 weeks (due to severe RA^4^) and therefore were excluded from the analysis.

As described earlier, all *BPSM1*^*+/+*^*TP53*^*−/−*^ mice had died by 8 months, with an average lifespan of 120 days. Most of these animals (~80%) had developed lymphoma, usually in the thymus. To our surprise, chronic inflammation due to constitutive overexpression of TNF did not influence tumour development elicited by p53 deficiency, and *BPSM1*^*+/m*^*TP53*^*−/−*^ mice died at the same average age and presented with the same tumour spectrum as *BPSM1*^*+/+*^*TP53*^*−/−*^ mice (~78% lymphoma, with 22% of mice killed due to paralysis) (*n*=23, 18 lymphomas) (Figure 1a). Our analysis also revealed that loss of p53 did not aggravate the bone erosion (Figure 1b) or the heart valve disease in BPSM1 mice. This contrasts with a report that loss of p53 exacerbated collagen-induced arthritis (CIA).^7^ This difference might be explained by the different mechanisms that elicit RA in the CIA *versus* the BPSM1 models. In the CIA model, disease is auto-antibody-mediated and therefore depends on both B and T cells. In contrast, in the BPSM1 model disease develops independently of B and T cells, but is driven by TNF. Perhaps some p53 effector processes, such as cell cycle arrest, apoptosis or repression of angiogenesis,^2^ limit tissue damage in the CIA, explaining why p53 loss would aggravate disease. Furthermore, the different genetic backgrounds of the mice used in the two studies (DBA1 for CIA *versus C*57BL/6 for BPSM1) might also explain the differences in impact of loss of p53.

All *BPSM1*^*+/m*^ mice become paralysed due to the severe bone erosion of T9–T13 vertebrae and have to be killed at around 200 days of age. Because high rates of p53 mutations have been found in the synovial tissue of rheumatoid arthritis patients,^8^ we predicted that loss of one allele of p53 might promote the neoplastic transformation of this tissue. However, *BPSM1*^*m/+*^*TP53*^*+*/−^ mice lived as long as *BPSM1*^*m/+*^*TP53*^*+/+*^ mice, and none presented signs of synovial or bone tumours by the time they became paralysed. Our results suggest that the chronic inflammation due to constitutive overexpression of TNF is not sufficient to significantly accelerate tumorigenesis driven by the loss of p53.

While all p53 mutations result in the loss of wild-type p53 activity, many are postulated to also result in the gain of a novel pro-oncogenic function. It remains possible that such *de novo* pro-oncogenic functions of mutant p53 might be needed to synergize with the chronic inflammatory state (for example, due to TNF overexpression) to accelerate tumorigenesis. This could be tested by crossing our BPSM1 mutant mice with p53 mutant mice.

## Figures and Tables

**Figure 1:**
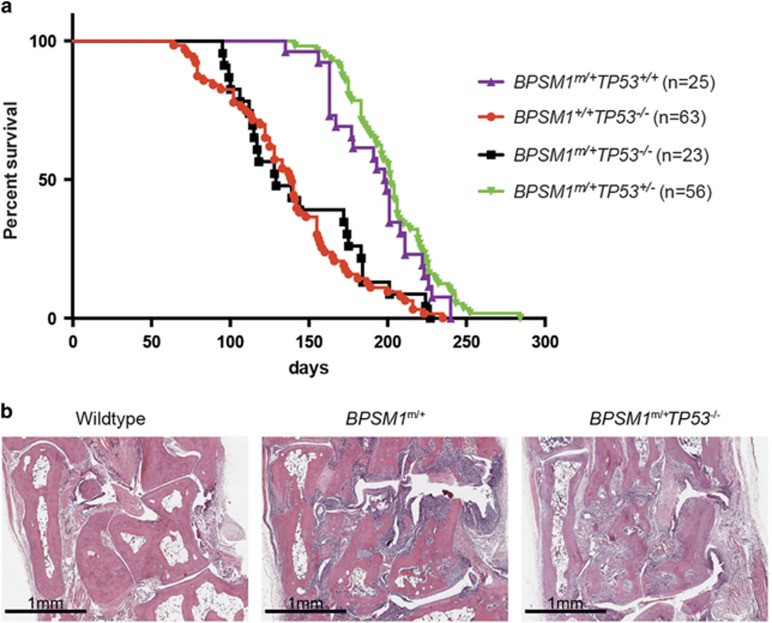
(**a**) Kaplan-Meier curve showing the early death of mice of the indicated genotypes. The BPSM1 mutation does not accelerate tumour development in p53-deficient mice (compare red and black curves), and TP53 heterozygosity does not accelerate the development of fatal RA in BPSM1 mutant mice (compare purple and green curves). (**b**) Representative haematoxylin and eosin-stained sections through the ankles of 100 day-old mice of the indicated genotypes, showing similar bone erosion and pannus invasion in *BPSM1*^*m/+*^*TP53*^*+/+*^ and *BPSM1*^*m/+*^*TP53*^*−/−*^ mice
